# Application of intraoperative frozen section examination in the management of female breast cancer in China: a nationwide, multicenter 10-year epidemiological study

**DOI:** 10.1186/1477-7819-12-225

**Published:** 2014-07-18

**Authors:** Ke Wang, Yu Ren, Rong Huang, Jian-Jun He, Wei-Liang Feng, Ya-Nan Kong, Feng Xu, Lin Zhao, Qing-Kun Song, Jing Li, Bao-Ning Zhang, Jin-Hu Fan, Xiao-Ming Xie, Shan Zheng, You-Lin Qiao

**Affiliations:** 1 Department of Oncosurgery, the First Affiliated Hospital of Medical College, Xi’an Jiaotong Universtiy, 277 Yanta West Road, Xi’an 710061, P.R. China; 2 Department of Cancer Epidemiology, Cancer Institute & Hospital, Chinese Academy of Medical Sciences & Peking Union Medical College, 17 South Panjiayuan Lane, Beijing 100021, P.R. China; 3 Department of Epidemiology, West China School of Public Health, Sichuan University, Chengdu, Sichuan 610041, P.R. China; 4 Department of Breast Surgery, Zhejiang Cancer Hospital, No. 38 Banshanqiao Guanji Road, Hangzhou 310022, P.R. China; 5 Department of Breast Oncology, Sun Yat-Sen University Cancer Center, 651 Dongfeng East, Guangzhou 510060, P.R. China; 6 Department of Breast-thyroid Surgery, Xiangya Second Hospital, Central South University, No. 139 Renminzhonglu, Changsha 410011, P.R. China; 7 Department of Breast Surgery, Liaoning Cancer Hospital, No. 44 Xiaoyanhe Road, Dadong District, Shenyang 110041, P.R. China; 8 Center of Breast Disease, Cancer Institute & Hospital, Chinese Academy of Medical Sciences & Peking Union Medical College, 17 South Panjiayuan Lane, Beijing 100021, P.R. China; 9 Department of Pathology, Cancer Institute & Hospital, Chinese Academy of Medical Sciences & Peking Union Medical College, 17 South Panjiayuan Lane, Beijing 100021, P.R. China

**Keywords:** Application mode, Female breast cancer, Intraoperative frozen section examination, Sociodemographic factor

## Abstract

**Background:**

Intraoperative frozen section examination (IFSE) during breast cancer surgery can partly reflect the status of surgical treatment since the surgical method used directly determines the purpose of IFSE use in disease management. This study aims to investigate the application of, changing trends in, and factors influencing IFSE in the management of female breast cancer in China.

**Methods:**

We collected the sociodemographic and clinical data of 4,211 breast cancer patients between 1999 and 2008 and statistically analyzed these data using χ^2^ or Fisher’s exact tests.

**Results:**

A total of 2,283 (54.22%) patients with breast cancer underwent IFSE. During the 10-year study period, IFSE use was associated with an increase in the number of sentinel lymph node biopsies (SLNB) and breast-conserving surgeries (BS) performed, with significant regional differences noted in this trend (*P* <0.05). Patients’ education, occupation, age, tumor size estimated by preoperative palpation, and the use of imaging examinations affected the purpose of IFSE use (*P* <0.05).

**Conclusions:**

Our results show that the purpose of IFSE in the surgical treatment of breast cancer in China is gradually approaching that in developed countries. We believe that policymakers must address the differences in breast cancer treatment based on the socioeconomic status of patients. Lastly, the use of IFSE for determining tumor characteristics should be avoided as far as possible, and patient education and breast cancer screening programs tailored to the Chinese population should be established. Our findings may guide the formulation of breast cancer control strategies in China and other low-income countries.

## Background

Intraoperative frozen section examination (IFSE) was first performed by William H. Welch in 1891. This method permits the rapid diagnosis of tumors during operation [1] and has become an indispensable part of surgical treatment of breast cancer, since it can guide operators in deciding the appropriate protocols during surgery [2]. Before the 1980s, IFSE was mostly used to determine the benignancy or malignancy of tumors intraoperatively [3]. Since 2000, with advances in tumor treatment methods as well as preoperative diagnostic methods, the use of IFSE has changed dramatically, especially in the case of breast cancer [4,5]. IFSE during breast cancer surgery can reflect, to a certain extent, the status of patients’ surgical treatment, since the surgical method used directly determines the purpose for which IFSE is used [6].

Although breast cancer is the most common cancer among women in China [7], few studies have analyzed the total population of breast cancer patients in the country. Our previous studies demonstrated, for the first time, the pathological characteristics of breast cancer in Chinese women [8,9]. The current study is part of a series of studies concerning the application and temporal tendencies of and regional differences in IFSE use in the diagnosis and treatment of female breast cancer in China during the period 1999–2008. We also examined factors affecting the purpose for which IFSE is used in breast cancer management.

## Methods

### Subjects

This series of studies was approved by the Institutional Review Board of the Cancer Foundation of China. The approval covered data collection from all the participating centers. Female patients with breast cancer who had received treatment (surgery, medical oncology, radiotherapy) were enrolled from seven first-class, grade III hospitals or referral centers in north, northeast, central, south, east, northwest, and southwest China [8]. Since there was no risk associated with participating in the study, patients’ informed consent was not obtained. The identity of all patients was kept anonymous.

### Data collection

According to the pre-designed recruitment protocol, at least 50 female patients with breast cancer were sampled from each hospital or referral center per year. The sampling method used could largely avoid selection bias. Two data input clerks from each site independently double-entered data from the patients’ record into a computer database. Quality control was performed during each step of data collection. Finally, all completed double-entry databases were sent to the Cancer Hospital/Institute, Chinese Academy of Medical Sciences, for validation using the statistical software EpiData ( http://www.epidata.dk/). Details of the methods have been described elsewhere [8].

Patient characteristics collected were general information, risk factors, imaging examination results, treatment mode, and clinicopathological characteristics of the tumors. Histological subtyping of tumors was based on the 1981 and 2003 WHO histological classification criteria [10,11]. Breast cancer staging was performed according to the American Joint Committee on Cancer tumor-node-metastasis staging system of 1997 and after [12,13].

### Grouping criteria

Subjects were assigned to two groups depending on the reason for using IFSE. In Group A, IFSE had been used for diagnosing the type of breast tumor, while in Group B, it had been used to assess metastasis to a sentinel lymph node (SLN) and/or to identify the resection margin during breast-conserving surgery (BS) but not to identify the type of primary tumor.

The seven geographic regions mentioned were classified as high- or low-socioeconomic status (SES) areas, according to previously described criteria [14]. Four socioeconomic indicators were recorded: gross domestic product per capita, percentage of health-service expenditure in the regional/provincial public affairs general budget, ratio of urban to rural population, and percentage of illiteracy among females aged 15 and over. They were used to establish the SES of each area with the selected hospital or referral center.

According to the commonly used classification in China, the ages of the patients at diagnosis of breast cancer were divided into four groups: <35 years, 35–49 years, 50–64 years, and >64 years.

### Statistical analysis

All statistical analyses were performed using the statistical software SPSS version 16.0 (SPSS Inc., Chicago, IL, USA). The trend χ^2^ test was employed to examine the temporal changes in IFSE application and its frequency during the period 1999 to 2008. Factors affecting the use of IFSE were analyzed using the χ^2^ test. The effect of preoperative examination methods on the frequency of IFSE use was evaluated using the χ^2^ test and Fisher’s exact test. A *P* value <0.05 was considered statistically significant.

## Results

### Patient characteristics

A total of 4,211 eligible breast cancer patients were enrolled in this series of studies, which comprised 9.3% of the total number of breast cancer cases encountered at the hospitals or referral centers [8]. Included in the 4,211 patients were 2,283 patients (54.22%) who had undergone IFSE. In terms of age, 139 patients (6.09%) were aged under 35 years at diagnosis, 1,145 (50.15%) were aged between 35 and 49 years, 793 (34.73%) between 50 and 64 years, and 206 (9.02%) above 64 years. As mentioned, the seven geographic regions were grouped into low- and high-SES areas according to the area-based SES. High-SES areas were north, northeast, east, and south China, with 1,227 subjects (53.75%); low-SES areas were northwest, central, and southwest China, with 1,056 subjects (46.25%).

### Temporal changes in the use of IFSE

**Figure 1:**
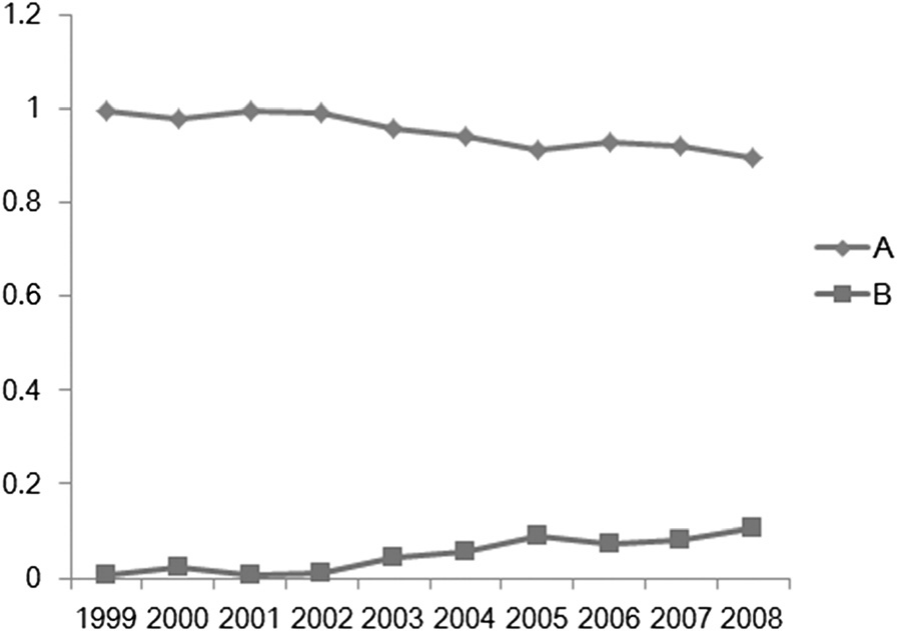
Temporal changes in the frequency of intraoperative frozen section examination performed for (A) identifying the type of primary tumor and (B) assessing metastasis to sentinel lymph nodes and/or identification of the resection margin.

The total number and percentage of the patients who underwent IFSE during the 10-year study period did not change significantly year-on-year, as shown in Table 1. There was, however, a change in the reason for using IFSE (Table 2 and Figure 1). The number and percentages of IFSEs performed for identifying the type of primary tumor declined gradually, while the corresponding figures for assessment of metastasis to SLNs or identification of the resection margin increased gradually (*P* <0.05). Overall, during the study period, 94.61% (2,160/2,283) of IFSEs were used to identify the type of primary tumor, 2.98% (68/2,283) for sentinel lymph node biopsy (SLNB), and 9.46% (216/2,283) for BS. In most cases, IFSE was used exclusively for identifying the type of primary tumor (2,014/2,283; 88.22%), whereas in 4.91% of cases (112/2,283), it was performed to identify both the type of primary tumor and the resection margin for BS; fewer cases involved IFSE use exclusively for SLNB or identification of the resection margin for BS. Thus, IFSE for identifying the type of primary tumor (Group A) accounted for 94.61% of cases (2160/2,283), while that for other reasons accounted for 5.39% of cases (123/2,283) (Group B; Table 2).

**Table 1:** Number and percentage of patients who underwent intraoperative frozen section examination (IFSE) between 1999 and 2008

**Year**	**IFSE (%)**	**No IFSE or unknown (%)**	**Total**
1999	210 (52.11)	193 (47.89)	403
2000	169 (48.29)	181 (51.71)	350
2001	199 (52.79)	178 (47.21)	377
2002	178 (52.20)	163 (47.80)	341
2003	238 (61.03)	152 (38.97)	390
2004	241 (57.79)	176 (42.21)	417
2005	202 (49.75)	204 (50.25)	406
2006	259 (56.06)	203 (43.94)	462
2007	334 (58.80)	234 (41.20)	568
2008	253 (50.91)	244 (49.09)	497
Total	2,283 (54.22)	1,928 (45.78)	4,211

**Table 2:** Frequency of intraoperative frozen section examination (IFSE) use between 1999 and 2008

**Year**	**Group A (%)**	**Group B (%)**	**Total**
1999	209 (99.52)	1 (0.48)	210
2000	165 (97.63)	4 (2.37)	169
2001	198 (99.50)	1 (0.50)	199
2002	176 (98.88)	2 (1.12)	178
2003	228 (95.80)	10 (4.20)	238
2004	227 (94.19)	14 (5.81)	241
2005	184 (91.09)	18 (8.91)	202
2006	240 (92.66)	19 (7.34)	259
2007	307 (91.92)	27 (8.08)	334
2008	226 (89.33)	27 (10.67)	253
Total	2,160 (94.61)	123 (5.39)	2,283

Group A: IFSE had been used for diagnosing the type of breast tumor.

Group B: IFSE had been used to assess metastasis to a SLN and/or to identify the resection margin during BS but not to identify the type of primary tumor.

### Comparison of regional differences in the frequency of IFSE

**Figure 2:**
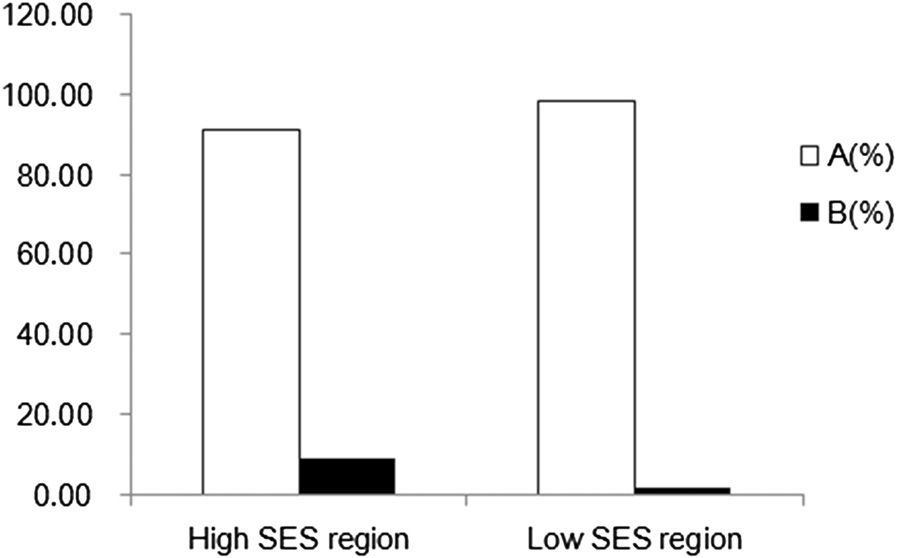
**Comparison of the frequency of intraoperative frozen section examination (IFSE) between low- and high-SES regions. (A)** IFSE use to identify the type of primary tumor. **(B)** IFSE use to assess metastasis to sentinel lymph nodes and/or identification of the resection margin.

The frequency of IFSE use for various reasons differed among the different SES regions as well. In low-SES areas, the percentage of IFSE used for assessment of metastasis to SLNs and/or identification of the resection margin (Group B) was very low (1.52%; 16/1,056). In contrast, in high-SES areas it was significantly higher (8.72%; 107/1,227; *P* <0.05) (Figure 2).

### Effect of sociodemographic characteristics on the frequency of IFSE use

Table 3 shows the effects of patients’ sociodemographic characteristics on indications for IFSE. Most patients who underwent IFSE were educated up to junior and senior high school (48.96%; 638/1,303), and the main occupation was manual work, accounting for 51.07% (1,024/2,005) of the total patient population. A little more than half the study population was in the 35 to 49 years age range (50.15%; 1,145/2,283). Statistical analyses showed that the level of education, occupation, and age all affected the indications for IFSE, as follows: i) Group A gradually reduced with advanced education level, while Group B increased significantly (*P* = 0.001). ii) The purpose of IFSE use also varied according to patients’ occupations. The highest percentage of Group A (96.09%) was observed among manual workers, while the highest percentage of Group B (8.37%) was found among intellectual workers, and the between-group difference was significant (*P* <0.001). iii) Lastly, Group A showed a gradually increasing tendency with age, while Group B exhibited a gradually declining tendency with age (*P* <0.001).

**Table 3:** Effect of patients’ sociodemographic characteristics on the frequency of IFSE use

**Sociodemographic characteristic**		**Group A**		**Group B**		**Total**	**χ**^**2 **^**value**	***P *** **value**
		**Number**	**Percentage (%)**	**Number**	**Percentage (%)**			
Education level	Primary school and lower	372	98.41	6	1.59	378		
	Junior and senior high school	626	98.12	12	1.88	638	14.835	0.001
	Junior college and higher	270	94.08	17	5.92	287		
Total		1,268		35		1,303*		
Occupation	Manual work	984	96.09	40	3.91	1,024		
	Intellectual work	569	91.63	52	8.37	621	15.902	<0.001
	Other**	344	95.56	16	4.44	360		
Total		1,897		108		2,005*		
Age (years)	<35	114	82.01	25	17.99	139	59.602	<0.001
	35–49	1,074	93.80	71	6.20	1,145		
	50–64	774	97.60	19	2.40	793		
	>64	198	96.12	8	3.88	206		
Total		2,160		123		2,283		

* Patients whose data were unavailable are not included in the table. ** Other: Those with occupations that are difficult to classify as manual or intellectual work, namely, housewives, merchants, soldiers, and unemployed individuals.

### Effects of preoperative examinations on purpose of IFSE use

The effects of preoperative examinations on the indications for IFSE are shown in Table 4. Both the tumor size, determined by preoperative palpation, and the performance of molybdenum-target X-ray or ultrasonography examinations prior to surgery affected the purpose of IFSE use (*P* <0.05). IFSE was performed in a higher percentage for Group B purpose in patients who had tumor sizes ≤20 mm or who had undergone molybdenum-target X-ray or ultrasonography examinations (*P* <0.05); on the contrary, the percentage of Group A was significantly lower in these patients (*P* <0.05). The lymph node status, determined by preoperative palpation, was not significantly correlated with the purpose of IFSE use (*P* >0.05).

**Table 4:** Effects of preoperative examinations on the frequency of intraoperative frozen section examination use

**Preoperative examination items**		**Group A**		**Group B**		**Total**	**χ**^**2 **^**value**	***P *** **value**	**Fisher’s exact probability**
		**Number**	**Percentage (%)**	**Number**	**Percentage (%)**				
Tumor size estimated by palpation	≤20 mm	752	93.07	56	6.93	808	5.842	0.016	–
	>20 mm	1,408	95.46	67	4.54	1,475			
Total		2,160		123		2,283			
Lymph node status determined by palpation	No metastasis (N0)	1,470	95.08	76	4.92	1,546	0.315	0.575	–
	Metastasis (N1–3)	566	94.49	33	5.51	599			
Total		2,036		109		2,145*			
Molybdenum-target X-ray	Not used	1,368	96.75	46	3.25	1,414	12.333	<0.001	–
	Used	655	93.44	46	6.56	701			
Total		2,023		92		2,115*			
Ultrasonography	Not used	612	97.45	16	2.55	628	6.832	0.009	–
	Used	1,421	94.92	76	5.08	1,497			
Total		2,033		92		2,125*			
MRI	Not used	2,083	95.16	106	4.84	2,189	–	–	0.088
	Used	65	90.28	7	9.72	72			
Total		2,148		113		2,261*			

* Patients whose data were not available are not included in the table.

## Discussion

With continual advances in science and technology and evolution of breast cancer treatment, IFSE plays an increasingly important role in the surgical treatment of this disease. Simultaneously, the principal purpose of IFSE has shifted from identification of tumor type to identification of SLN metastasis and assessment of the resection margin during BS. In our previous nationwide multicenter retrospective clinical epidemiological studies of female breast cancer in China, we described the pathological characteristics of this condition [8,9]. The present study, which is part of a series of studies, investigated the application of IFSE and factors that influence its use in the management of female breast cancer. An objective of this study was to guide the formulation of breast cancer control strategies in China and other low-income countries.

First, we analyzed the indications for IFSE and changing trends in its use. The results showed that IFSE was still mainly used to identify the type of breast tumor. However, its overall use for this purpose has declined over the 10-year study period, while its use for assessment of metastasis to SLNs and identification of the resection margin for BS has gradually increased. The indications for IFSE in China differ from those in developed countries, where SLNB and BS are the major indications for surgical treatment of breast cancer [15-27]. Correspondingly, the purpose of IFSE use has changed. IFSE is increasingly being applied in SLNB and BS and less so for identifying the type of primary tumor [15]. Both the Edge report from the 28^th^ Annual San Antonio Breast Cancer Symposium [28] and the results of the NSABP B-32 clinical trial [29] indicated that open surgical resection combined with IFSE is no longer an acceptable treatment for breast cancer in developed countries. However, differences in economic levels may necessitate differences in treatment modes for breast cancer across countries [30-32]. The overall level of economic development is significantly lower in China than in developed countries, and this could affect disease-control strategies. In the present area-stratified analysis, we found that regional differences in economic development affected the use of IFSE: regions with high-SES tended to use IFSE for assessment of metastasis to SLNs and identifying the resection margin during BS more frequently than those with low-SES. We believe that in developing countries or regions, the use of preoperative biopsy techniques should be increased in order to decrease the use of IFSE for intraoperative diagnosis of breast tumor so that we can choose the more appropriate treatment pattern before operation. Our results are encouraging in that we found that the use of IFSE for SLNB and BS has significantly increased over the 10-year study period, while its use for identifying the type of primary tumor has decreased. This trend suggests that surgical treatment of breast cancer in China is gradually approaching that of developed countries. We believe that the influence of regional differences in economic levels on the use of IFSE and breast cancer control strategies deserves more attention as a means of benefiting more patients.

We also evaluated the effects of patients’ sociodemographic characteristics on the use of IFSE for surgical treatment of breast cancer. We found that the purpose of IFSE use was closely associated with patients’ education, occupation, and age. This association may be attributed to the following factors: i) The time to diagnosis is longer in the case of patients of lower educational level, lower occupational income, and older age [33-35], because of which they have advanced disease and the opportunity to perform SLNB and BS is poor. ii) Subjects with higher educational levels and income are more willing to accept new concepts and technologies, such as SLNB and BS [36,37]. iii) Subjects with lower incomes cannot afford post-surgical radiotherapy, and most live in poor areas where radiotherapy is seldom performed [37]. iv) Younger patients are more likely to care about the esthetics of surgery and hence may be more willing to accept BS and SLNB [38,39], while older patients are more prone to reject these two types of surgery [40]. On the basis of these findings, we believe that better education among individuals of low-SES should be emphasized in order to help them accept SLNB and BS.

Finally, we assessed the effects of preoperative examinations on the purpose of IFSE use. Our previous study showed that imaging examinations affected the clinicopathological characterization of breast cancer at diagnosis [9]. The current study found that the frequency of imaging examinations also affected the rate of IFSE use. Greater implementation of imaging examinations led to greater use of IFSE for SLNB and BS. The following reasons may explain this phenomenon: i) Breast cancer can be detected at earlier stages in patients who have undergone molybdenum-target X-ray and ultrasonographic examinations; therefore, their tumors may be smaller and the possibility of SLN metastasis is lower [41,42], and, consequently, these patients are more likely to receive SLNB and BS. ii) Second, preoperative molybdenum-target X-ray and ultrasonographic examinations can better predict the safety of SLNB and BS [43-45]. In the present study, we also found that tumor size, as estimated by preoperative palpation, affected the indications for IFSE, in that IFSE was used more frequently for SLNB and BS in patients with tumors ≤20 mm. This finding may demonstrate that preoperative estimation of tumor size is a key factor in determining whether BS will be performed [46,47], as BS is safest when tumors are small [48]. Conversely, patients with large tumors have a higher probability of having axillary lymph node metastasis [49,50] and a greater risk of complications from a SLNB [51,52].

Our study has a limitation, which must be acknowledged: since subjects were enrolled from hospitals or referral centers in seven traditional geographic regions, a selection bias may have been present. In China, most patients with breast cancer visit high-grade hospitals. All hospitals or referral centers involved in the present study are grade III and have the requisite physician resources and equipment for performing IFSE. However, grade I and II hospitals lack the resources to perform comprehensive breast cancer therapy, including IFSE. Nonetheless, since our study had a broad base and included diverse populations, the selection method used is probably the most suitable to reflect the diverse nature of present-day China.

## Conclusions

In summary, in the present study, we analyzed the use of IFSE in the diagnosis and treatment of breast cancer in China during the 10-year period from 1999 to 2008. From our observations, we concluded that IFSE is still used mainly to identify the type of breast tumor. Ideally, the use of preoperative biopsy techniques should be improved so that IFSE use for tumor diagnosis during breast cancer operations can be decreased. In line with the introduction of newer concepts in breast cancer treatment and treatment modes, the purpose of IFSE in surgical treatment of breast cancer in China is gradually approaching that of developed countries. Nevertheless, regional differences in IFSE use require the attention of policymakers. Finally, patients’ sociodemographic characteristics and the availability and use of preoperative imaging examinations significantly affect the use of IFSE in breast cancer treatment. Therefore, better education and breast cancer screening suitable for China’s needs may help prevent breast cancer and aid the system in serving those with this disease.

## Abbreviations

BS: Breast-conserving surgery; IFSE: Intraoperative frozen section examination; SLNB: Sentinel lymph node biopsy; SLN: Sentinel lymph node; SES: Socioeconomic status.

## Competing interests

The authors declare that they have no competing interests.

## Authors’ contributions

KW and YR helped to analyze and interpret the data and drafted the initial manuscript. RH, JJH, YR, and WLF helped to design the study. WLF, YNK, FX, LZ, and QKS helped with local data collection. JL, BNZ, and JHF helped with the data management and analysis. XMX and SZ did critical revisions of the manuscript. All authors read and approved the final manuscript.
